# Nutritional Strategies to Mitigate Heat Stress in Cattle: A Narrative Review

**DOI:** 10.3390/microorganisms14071511

**Published:** 2026-07-10

**Authors:** Rajan Dhakal, Volker Krömker, Michael Van Amburgh, Niels Moritz, Christine Brøkner, André Luis Alves Neves, Svenja Woudstra

**Affiliations:** 1 Department of Veterinary and Animal Sciences, University of Copenhagen, DK-1870 Frederiksberg, Denmark; volker.kroemker@hs-hannover.de (V.K.); andre.neves@sund.ku.dk (A.L.A.N.); 2 VILOFOSS, Ballesvej 2, DK-7000 Fredericia, Denmark; 3 Department of Bioprocess Engineering-and Microbiology, University of Applied Sciences and Arts, D-30453 Hanover, Germany; 4 Department of Animal Science, Cornell University, Ithaca, NY 14853, USA; 5 Deutsche Vilomix Tierernährung GmbH, Bahnhofstraße 30, D-49434 Neuenkirchen-Vörden, Germany

**Keywords:** nutritional intervention, feed additive, diet, nutrition management, oxidative stress, sensor technology, in vitro

## Abstract

Heat stress is a growing concern in cattle production systems due to the increasing frequency and intensity of extreme weather events driven by climate change. This review synthesizes current knowledge on the multifaceted impacts of heat stress and focuses on nutritional strategies to mitigate its effects on ruminating cattle. A comprehensive literature search was conducted using PubMed, Scopus, Web of Science, and Google Scholar. Heat stress adversely affects cattle physiology, behavior, rumen function, and overall productivity, particularly in dairy animals with high metabolic activity. During heat stress episodes, changes in the microbial population have been reported; however, there is no clear consensus, as findings vary widely among studies depending on diet, feed intake, animal type and experimental design. This variability limits the ability to draw general conclusions regarding changes in the rumen microbiome driven by heat stress. In this context, dietary nutritional intervention strategies offer a practical and scalable approach to enhance thermotolerance and maintain performance under heat stress conditions. Key nutritional strategies include modifications in diet composition to reduce metabolic heat production, with some approaches carrying potential risks to animal health, e.g., increasing dietary energy density through concentrates while minimizing forage content. Supplementation with rumen-protected nutrients like amino acids, vitamins, and minerals can be used to support immune function, antioxidant capacity, and metabolic stability. Polyphenols and betaine contribute to oxidative stress reduction and gut integrity, while probiotics may be used to improve rumen fermentation and nutrient utilization. Sensor technologies, including rumen boluses and wearable devices, offer the potential to monitor physiological responses to heat stress in real time and offer opportunities for precision feeding and early intervention. Most published studies only cover short periods of heat stress, and there is a lack of in vitro models simulating rumen hyperthermia. In parallel, future research should therefore prioritize longitudinal, in vivo trials that integrate physiological, metabolic, and microbial responses to understand the long term and systemic effect of heat stress. In addition, controlled trials in commercial settings are necessary to prove the transferability of results to commercial herds. A multidisciplinary approach combining nutritional, environmental, and technological strategies is likely to play an important role in safeguarding cattle welfare and productivity in a warming climate.

## 1. Introduction

**Figure 1:**
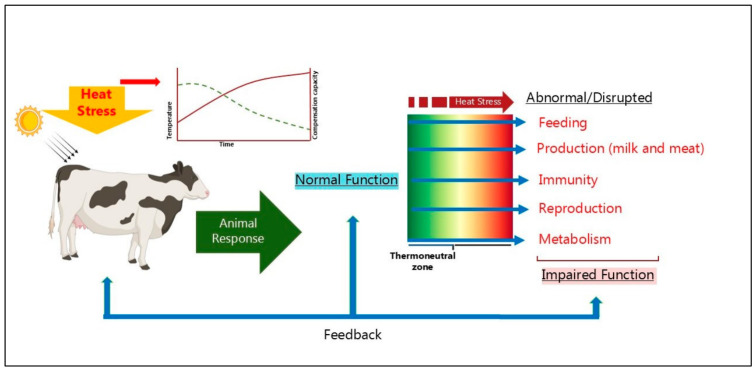
A schematic representation of animal responses to heat stress.

The rising global temperatures have far-reaching consequences on our planet. One of the most immediate impacts is the increasing frequency and intensity of extreme weather events, such as heat waves, storms, and heavy rainfall. Planet surface temperatures have been predominantly warmer than average since the mid-20th century, and the overall trend indicates an increase of +0.10 °C per decade [1]. These shifts in temperature patterns have been associated with an increased frequency and intensity of heat stress in livestock, which is a significant concern in both intensive and extensive production systems. Heat stress is a condition where the animal’s body cannot dissipate excess heat due to environmental factors especially high ambient temperatures and high humidity, as well as factors such as high solar radiation, low wind speed, and a lack of shade and ventilation [2]. These conditions lead to physiological and behavioral changes that can negatively impact health and productivity (Figure 1) [3,4]. Dairy cattle are particularly susceptible to heat stress due to their high dry matter intake and increased metabolic activity [5]. Dairy producers and nutritionists, therefore, often increase dietary energy density through increased concentrate feeding to support energy intake and improve glucose status under heat stress [6]. However, this strategy can increase the risk of subacute ruminal acidosis if the amount of physically effective fiber is insufficient. To encourage the adoption of less risky feeding strategies, this review discusses the current knowledge about nutritional strategies to mitigate heat stress in ruminating cattle.

This review follows a structured narrative approach. A comprehensive literature search was conducted using PubMed, Scopus, Web of Science, and Google Scholar to identify studies addressing heat stress, rumen physiology, and nutritional mitigation strategies in cattle from 1980 to March 2026. Representative Boolean combinations included (‘heat stress’ OR ‘thermal stress’) AND (‘cattle’ OR ‘dairy cows’) AND (‘nutrition’ OR ‘rumen microbiome’), with additional improvement using specific filters (e.g., animal type, experimental design, and publication type). Studies were selected based on relevance rather than a formal systematic review protocol. Only peer-reviewed English-language articles were included. Primary emphasis was placed on cattle studies; evidence from other species was included only where mechanistically relevant. Further, studies were first screened by title and abstract, followed by full-text evaluation to extract information on physiological and metabolic responses to heat stress, rumen microbial changes, detection technologies, and nutritional strategies to improve thermotolerance in cattle. This review integrates rumen microbiome dynamics under rumen hyperthermia and emerging in vitro fermentation models to provide a mechanistic framework for nutritional interventions in cattle exposed to heat stress.

## 2. Effects of Heat Stress on Animal Physiology

Heat stress exerts both acute and chronic effects on dairy cattle physiology through distinct but interacting mechanisms [7]. In pregnant cows, effects have been described to go beyond the impact on the dam, also impacting the unborn calf through placental dysfunction and epigenetic modification. This section focuses on the known effects of heat stress on cattle, as nutritional intervention strategies discussed in this review are primary to this group of animals.

Heat-stressed cattle exhibit several behavioral changes [8]. In heat-stress conditions, cattle tend to reduce feed intake and alter feeding times to cooler parts of the day, such as early morning or late evening, when core body temperature has decreased to the point that encourages feed intake [9]. To avoid sunlight during heat stress, cattle will spend more time in shaded areas and may stand more to increase heat dissipation, potentially leading to lameness [8]. Social behaviors also change, with increased aggression and competition for resources such as shade and water. Heat stress reduces productivity in both dairy and beef cattle; however, the outcomes differ substantially. In dairy cattle, heat stress can reduce milk yield and quality (i.e., fat and protein content) due to decreased feed intake and metabolic changes [10]. Also, in beef cattle, reduced feed intake, increased energy expenditure for cooling, and increased stress hormones due to heat stress can result in slower growth rates and lower meat quality [11]. Reproductive performance is negatively affected by heat stress, resulting in lower conception rates, increased embryonic loss, and irregular estrous cycles in both dairy and beef cattle [12,13]. These differences reflect distinct metabolic priorities and should be considered when evaluating mitigation strategies.

Cattle under heat stress initiate compensatory changes in physical, biochemical, and biological pathways. Convection and evaporation are the major methods of heat loss in ruminants, and evaporation is the major mode of heat loss in dairy animals [14]. Although evaporation efficiency increases with rising temperature, high humidity in combination with high temperature limits heat loss through evaporation. To cope with heat stress, cattle increase the rates of respiration to increase evaporation. This increase in respiration rate changes the blood gas parameters during hot daytime hours [15]. There is the possibility of increases in the blood pO_2_ and decreases in the blood pCO_2_; these changes may shift the acid–base balance toward respiratory alkalosis [6,16].

**Figure 2:**
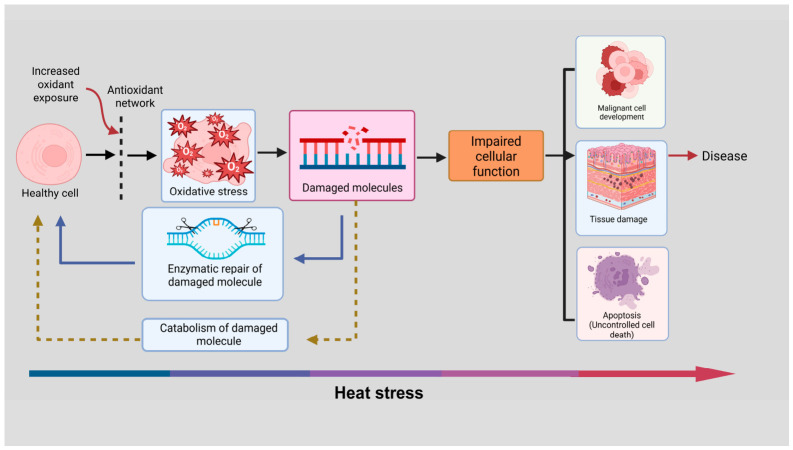
Schematic outline of oxidative stress-mediated cellular damage and progression due to heat stress to disease.

During short-term exposure to heat, cows increase their heart rate to increase peripheral blood flow, thereby increasing cardiac output [17]. In combination with surface vasodilatation, this increases the blood flow to the skin to achieve cooling. At the same time, this mechanism deprives the visceral blood flow (body core) [18], which may increase the production of free radicals or reactive oxygen species (ROS), and decrease antioxidant status due to ischemia/hypoxia [19]. In normal conditions, the production of ROS and neutralization is well balanced [20]. During ischemia caused by heat stress, the production of free radicals is faster than their neutralization by the antioxidant system. This leads to damage of macromolecules, disruption of normal metabolism and physiology, and may ultimately lead to loss of cell function [21]. The prolonged exposure to heat stress results in chronic heat stress, leading to sustained physiological adaptations and long-term effects on productivity and health. When heat-stress compensation mechanisms are overburdened, immune function declines, leading to increased intramammary infections and higher somatic cell count [22]. Prolonged heat stress also impairs performance and immunity [23]. Prolonged exposure to heat stress decreases blood glutathione (reduced form), increases oxidized glutathione concentration, and results in oxidative stress [24]. Cells manage oxidative stress in normal conditions through enzymatic repair and catabolism [25]. However, prolonged heat stress alters the damage-to-repair ratio (Figure 2). If delayed, the recovery from oxidative stress leads to impaired cellular function, tissue damage (apoptosis), and disease [26].

As the heat stress prolongs, animals will continuously show reduced feed consumption, reducing production for longer periods. During the 7-day short-term heat-stress period evaluated by Wheelock et al. [27], lactating Holstein cows experienced reduced dry matter intake and negative energy balance. To distinguish between the effects of reduced feed intake and heat stress per se, studies using thermoneutral pair-fed controls have shown that basal plasma non-esterified fatty acid (NEFA) concentrations did not increase in heat-stressed cows compared with thermoneutral pair-fed controls, suggesting that adipose tissue mobilization is not proportionally elevated despite negative energy balance. Additionally, plasma glucose decreases, insulin remains unaffected, and plasma urea nitrogen (PUN) increases [23]. The increase in PUN enhanced amino acid deamination and protein catabolism, suggesting that gluconeogenesis relies primarily on glucogenic amino acids rather than on adipose tissue mobilization. Together, these findings highlight that metabolic shift responses to heat stress cannot be explained solely by reduced feed intake, but also reflect heat-specific physiological adaptations that alter substrate partitioning, favoring amino acid catabolism over lipid mobilization to support glucose homeostasis [6].

Furthermore, heat stress during gestation can induce epigenetic programming effects that persist throughout life, including alterations in growth trajectories, mammary development, and anabolic capacity [28,29]. These programming effects may limit the magnitude of recovery from subsequent heat challenges. Late-gestation heat stress has a carryover effect, which lasts at least for two generations, and this results in a decrease of 2.2 kg milk/day in the F1 generation and 1.8 kg milk/day in the F2 generation compared to calves not experiencing heat stress during fetal development of their mothers [30]. In addition, heat stress can alter the methylation profile of liver and mammary DNA. It has been demonstrated that this can cause morphological alterations in the offspring during postnatal life, resulting in smaller and more compact alveoli. In addition, it impacts genes related to innate immune defense, mammary gland cell signaling, development, and transcription and translation [31,32]. The calves born from heat-stressed mothers are less likely to survive and this results in substantial increases in youngstock rearing costs.

In addition, heat stress disrupts the rumen ecosystem. The rumen functions as a large fermenter, generating substantial metabolic heat during the microbial breakdown of structural and non-structural carbohydrates. Under heat-stress conditions, ruminal heat production adds to the animal’s thermal burden, making it more difficult to maintain thermal homeostasis. Upon cell death of gram negative bacteria, they release lipopolysaccharides (LPS), which can compromise liver function and the blood–milk barrier, ultimately contributing to elevated somatic cell counts (SCC) in milk [33,34,35].

So, influencing rumen fermentation has emerged as a promising strategy to mitigate heat stress in cattle. One common approach adopted by nutritionists is to increase the proportion of concentrate feeds in the diet. This strategy aims to reduce the metabolic heat load associated with structural carbohydrate fermentation and to compensate for reduced feed intake during periods of heat stress. However, while concentrate-rich diets can help sustain energy intake and production, they are also associated with a lower rumen pH and an increased risk of rumen acidosis [36].

## 3. Technologies to Detect Heat Stress in Ruminants

Bioclimatic thermal indices are the most widely used tool to detect and predict heat stress in ruminants [37]. Relative humidity and temperature are predominant predictors in most bioclimatic thermal indices, such as the temperature–humidity index (THI) and heat load index (HLI). The THI and HLI are the two most used weather-based indices for monitoring cattle heat stress at the commercial level. Typically, THI above 65 is considered heat stress in cattle. However, THI does not account for other factors like solar radiation, wind speed, core body temperature, and respiration rate. To overcome the limitations of THI, researchers from around the world have developed and improved heat-stress predictors. Several alternative indices have been developed to improve the assessment of heat stress and they are as follows: (1) the black globe humidity index which accounts for solar radiation; (2) the equivalent temperature index, which accounts for cows net heat gain and reduction in milk production; and (3) the effective temperature index for dairy heifers, which predicts respiration rate and mean skin temperature by accounting for air and black globe temperature (BG) measures radiant heat, solar radiation and air movement. In addition, the heat load index currently used by the Australian feedlot industry accounts for BG, RH, and wind speed; and the equivalent temperature index for dairy cattle, which accounts for air temperature, relative humidity, air velocity, and solar radiation and their interactions [38,39,40,41,42]. All these indices are calculated from instantaneous environmental measurements (e.g., temperature, humidity, radiation, and wind speed) and therefore reflect conditions at a specific point in time rather than cumulative heat load.

Threshold values of THI for defining heat stress are not universal and vary depending on factors such as breed, production level, physiological stage, management system, and environmental conditions [43]. High-producing dairy cows may exhibit reduced performance at relatively low THI values, whereas beef cattle or more heat-adapted breeds may tolerate higher thresholds [44,45]. Given this variability, reliance on THI alone may not adequately capture the animal’s physiological response to heat stress [40]. Consequently, new technologies and increased computational power are rapidly improving the monitoring and prediction of heat stress in cattle by analyzing physiological responses such as respiration rate, skin temperature, and core body temperature [38]. Recent advances in precision livestock farming have enabled the integration of sensor data with machine learning algorithms for early detection and prediction of heat stress [46]. Machine learning models can process large datasets derived from wearable sensors, environmental measurements, and production parameters to identify complex patterns associated with heat stress. These approaches allow real-time monitoring and predictive modeling, improving decision-making in herd management [47]. For example, classification algorithms have been used to detect heat-stress events based on respiration rate, activity, and body temperature patterns, while predictive models can forecast heat-stress risk under changing environmental conditions [48]. Indications of heat stress can be measured using different commercially available sensor technologies installed on or inside the animal. Rumen-reticular boluses are one example. These devices can measure parameters such as temperature, acceleration, and activity, and may be used to estimate additional variables, including drinking behavior and respiration rate, through algorithm-based calculations rather than direct measurement [37,49,50,51]. Rising core body temperature in the absence of disease (i.e., fever) represents the stage of heat stress where the animal can no longer fully mitigate rising ambient temperatures [37,49,51]. Rumen temperature bolus appears to have the potential to detect these increases in body temperature, as they are also able to detect fever (e.g., in the case of bovine respiratory disease) [52,53].

## 4. The Rumen Microbiome and Heat Stress

The reticulo-rumen is the primary site of fiber degradation in the digestive tract of ruminants. It harbors a diverse and complex population of microorganisms, including bacteria, protozoa, fungi, viruses, and archaea. These microorganisms play a crucial role in breaking down fibrous plant material, particularly cellulose and hemicellulose, transforming the biomass into volatile fatty acids, vitamins, and microbial protein through fermentation processes that the animal can absorb, digest, and metabolize.

To date, most studies investigating the effect of heat stress on rumen fermentation have focused on changes in the microbiome. In ruminants, a core microbiome is found across a wide geographical range, but the complete rumen microbial community composition varies with diet and host [54]. Bacteroidetes, Firmicutes, and Proteobacteria are the three most dominant phyla in the rumen [55,56].

The effects of heat stress on the rumen microbiome are difficult to interpret, as observed changes may result from direct thermal effects, reduced feed intake, or diet composition adjustments during heat stress. The rise in environmental parameters such as temperature and humidity has a significant effect on the bacterial composition in the rumen [57]. Seasonal changes can alter rumen microbiota composition, structure, and fermentation characteristics—even when the same total mixed ration is fed during winter, spring, and summer [58]. Islam et al. [58] observed that the relative abundance of Bacteroidetes was significantly higher in spring and summer, while that of Firmicutes was higher in winter when fed with the same total ration. Because dry matter intake and feeding behavior often change under heat stress, some of these microbial shifts may be intake-mediated rather than driven directly by temperature.

Evidence from buffalo studies indicates similar directional shifts in certain microbial taxa under heat stress; however, these findings should be interpreted cautiously, as species-specific differences in rumen physiology and adaptation may limit direct extrapolation to cattle. Wang et al. [59] found that *Lactobacillales*, *Streptococcus*, *Leuconostocaceae*, and *Leissella* were significantly more abundant in the rumen of buffaloes under thermoneutral conditions than those experiencing heat stress. However, the richness and diversity of the microbial community were not affected. In contrast, in another study for some cellulolytic/hemicellulolytic bacteria (*Ruminococcus albus*, *Ruminococcus flavefaciens*) enrichment was observed [60]. After 6 days of short-term acute heat-stress exposure, Baek et al. [61] found that the relative abundance of fibrolytic *Ruminococcaceae* decreased while that of lactate-producing *Lactobacillaceae* and amylolytic *Prevotella* and *Ruminobacter* increased. Similarly, in lactating dairy cows, heat stress significantly increased the relative abundance of Bacteroidota (primarily gram-negative bacteria) while decreasing that of Firmicutes (primarily gram-positive bacteria) [34]. Although Bacteroidota are generally gram negative and Firmicutes are largely gram positive, these broad taxonomic classifications should be interpreted cautiously, as functional traits such as lipopolysaccharide production and inflammatory potential cannot be reliably inferred at the phylum level.

In summary, it appears that heat stress in cattle increases the abundance of lactate-producing bacteria, *Streptococcus*, unclassified Enterobacteriaceae, cellulolytic bacteria, Ruminobacter, Treponema, and unclassified Bacteroidaceae, Fibrobacteres, and decreases acetate-producing bacteria, Actinobacteria, and *Acetobacter* [62]. There is no clear consensus on changes in the rumen microbiome associated with heat stress (Table 1), with different studies reporting divergent or even contradictory findings. Current evidence on the effects of heat stress on the rumen microbiome remains inconsistent. This variability likely reflects substantial differences among studies in experimental design, including the severity and duration of heat-stress exposure, nutrition, animal species, breed, and production stage. These changes could be attributable not only to climatic conditions [34] but also to dietary changes [63], interventions during heat stress [64], and species, type, or breed [65,66,67]. As a result, establishing a consistent microbiome signature of heat stress in cattle remains challenging.

**Figure 3:**
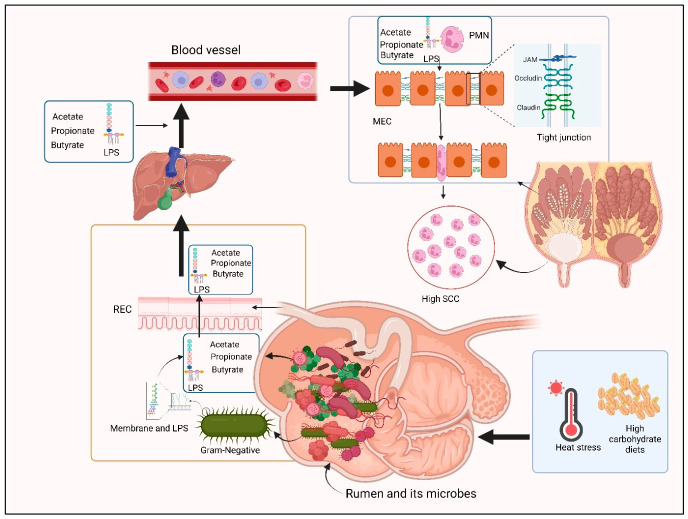
A schematic representation of the potential effects of heat stress on rumen function, illustrating how disruptions in the rumen microbiota may lead to increased release of lipopolysaccharides (LPS). These endotoxins may translocate across the rumen epithelium and are hypothesized to contribute to systemic inflammation, potentially affecting liver function and the integrity of the blood–milk barrier, which may be associated with increased somatic cell count (SCC) in milk, particularly under heat stress and high-carbohydrate diets. (LPS: Lipopolysaccharide, REC: Rumen epithelial cells, MECs: Mammary gland epithelial cells, and PMN: Polymorphonuclear).

Dysbiosis of the rumen has consequences for the host health and wellbeing. Patra et al. [70] reported that some alterations in the rumen microbiome during heat stress may be attributable to reduced feed intake rather than direct effects of elevated temperature, although acute heat stress can also induce microbiome changes independent of feed intake. An increase in lactate-producing microbes can be associated with ruminal acidosis [71]. Whereas the increase in the abundance of gram-negative microbes can increase lipopolysaccharide (LPS) in the rumen fluid after the lysis of their bacterial cells (Figure 3)**.** Under certain conditions, elevated rumen-derived LPS has been suggested to compromise rumen epithelial integrity, potentially allowing translocation into the bloodstream and affecting liver function [33]. Once in circulation, LPS may contribute to systemic inflammatory responses, which have been proposed to influence the mammary gland health; however, this pathway is complex and not fully established in heat-stressed cattle. Although associations between systemic inflammation and increased susceptibility to mastitis have been reported, a direct causal link between rumen-derived LPS and mastitis remains uncertain [33,72]. Some cattle breeds are more efficient in regulating body temperature than others [73], and the rumen microbiome can be more sensitive to heat stress in one breed than in another [67], suggesting that the rumen mechanism may differ in breeds adapted to heat stress.

Despite growing interest in understanding the rumen microbiome under heat stress [62], there is a shortage of published in vitro studies. This limits our ability to isolate microbial responses from host-related physiological effects and hinders the development of targeted nutritional interventions. Standardized in vitro methods are therefore necessary to simulate and assess the direct impact of elevated temperatures on microbial community composition and fermentation dynamics. The in vitro rumen fermentation method is the most commonly used experimental design to test the effect of feed and feed additives on rumen fermentation in ruminant research; however, a clear definition of physiologically relevant rumen temperature ranges that reflects heat stress is still lacking. Under normal conditions, rumen temperature in cattle typically ranges from approximately 38.5 °C to 39.5 °C, while mild increases during heat stress may raise rumen temperature to around 40–42 °C. Experimental incubation temperatures within this range can therefore be considered physiologically relevant for simulating rumen hyperthermia. In contrast, higher incubation temperatures (e.g., ≥ 43 °C) may represent more extreme or artificial conditions that are less representative of in vivo situations.

A defined protocol is needed to simulate heat stress in static, semi-dynamic, and dynamic in vitro rumen fermentation systems. Recent research on in vitro rumen fermentation at different temperatures reveals mixed results with complex interactions among temperature, diet composition, and microbial activity. Jo et al. [74] found that higher incubation temperature (41 °C) generally increases ammonia and volatile fatty acid production while decrease the abundance of liquid-associated bacteria, i.e., bacteria present in the rumen fluid fraction. Conversely, An et al. [75] found that severe hyperthermal conditions (42 °C) can reduce short-chain fatty acid production and alter microbial abundance.

These findings highlight that moderate increases in temperature may alter fermentation dynamics without suppressing microbial activity, whereas more extreme temperatures can impair microbial function. Therefore, careful selection of incubation temperature is critical when designing in vitro models to ensure physiological relevance.

## 5. Nutritional Strategies to Mitigate Heat Stress

While several mitigation strategies aim to directly influence external factors causing heat stress, e.g., lowering the environmental temperature or THI, or decreasing direct exposure to sunlight, other measures support animals through improved heat dissipation through evaporation. Finally, nutritional interventions are another part of advanced heat-stress management.

### 5.1. Adjustments in Feed Composition

Total heat from both environmental and metabolic heat on dairy cows is likely to increase over summer in grazing cattle or when fresh grass constitutes a significant part of the diet. High summer temperatures are intensified under these conditions by diets getting even more fibrous as grass ages and dry pastures. During heat stress, lactating cows reduce feed intake and therefore enter a negative energy balance [76]. Thus, one dietary intervention during heat stress aims to ameliorate this energy deficit by providing high-energy diets using increased concentrate or fat supplements [6]. However, these strategies pose challenges. High-concentrate diets can increase the risk of rumen acidosis due to reduced relative and absolute fiber intake (potentially falling below minimum fiber intake to sustain physiological rumen function). This leads to altered fermentation patterns, potentially compromising rumen health and microbial stability [77]. Moreover, reliance on energy-dense feeds may raise concerns about long-term sustainability and feed cost, especially in systems where forage-based diets are more economically and environmentally viable and feeding of concentrates increases competition with human food.

In addition to increasing dietary energy density, maintaining an appropriate fiber balance is essential during heat stress. Physically effective fiber (peNDF) plays a key role in stimulating rumination and saliva production, which are critical for buffering rumen pH [78]. Reductions in forage inclusion or particle size, often used to increase energy density, can compromise rumen function and increase the risk of subacute ruminal acidosis. However, fermentation of fibrous feeds (forages) generates significant metabolic heat, which can increase the animal’s thermal load under heat stress; thus, fiber supplementation represents a trade-off and must be carefully balanced to support rumen function without exacerbating heat stress [79,80]. Therefore, strategies to enhance energy intake must be carefully balanced with adequate fiber provision to maintain rumen stability.

Feeding management strategies can also play an important role in supporting intake and reducing heat-stress impacts. Providing feed during cooler periods of the day, such as early morning or late evening, can help maintain dry matter intake when ambient temperatures are lower [81,82]. Additionally, frequent feed delivery and maintaining fresh, palatable feed can encourage intake under heat-stress conditions. Water availability is another critical factor, as heat-stressed cattle exhibit increased water intake to support thermoregulation [83]. Ensuring continuous access to clean, cool water helps maintain hydration status and supports feed intake and rumen function [84]. In high-concentrate feeding systems or under conditions of reduced physically effective fiber, the use of dietary buffers may help maintain ruminal pH within the physiological range (approximately 5.8–6.5) by enhancing buffering capacity [85]; however, their effectiveness can vary depending on diet composition and intake patterns. Overall, while adjustments in feed composition and feeding strategies can support intake and rumen function during heat stress, these approaches primarily help maintain productivity rather than directly reducing physiological heat load.

### 5.2. Rumen Bypass Fat, Amino Acid, and Mineral Supplementation

Nutritional interventions applied during heat stress can serve two distinct roles: (1) supporting productivity by improving energy balance and metabolic efficiency, and (2) directly mitigating physiological heat load by reducing body temperature, influencing respiration rate, or oxidative and inflammatory responses. These outcomes are not equivalent, and improvements in production performance should not be interpreted as evidence of reduced thermal load without corresponding physiological measurements.

Microbes in the rumen ferment feed before it reaches the small intestine. Any additional nutrient supplements—such as fats, amino acids, and vitamins—need to be rumen-protected, as fats can disrupt rumen fermentation, and amino acids, micronutrients, and vitamins are often utilized by microbes, destroyed, or altered before the host can benefit from them.

#### 5.2.1. Rumen Bypass Fat Supplementation

Among them, rumen bypass fat supplementation is a strategy to improve energy balance in dairy animals, particularly during the transition from the dry period to lactation [86]. Rumen bypass fat supplementation has shown positive effects on dairy cattle performance, particularly under heat-stress conditions. Studies have reported increased milk production, fat-corrected milk yield, and milk fat percentage in cows supplemented with bypass fat [87,88,89]. Similarly, Wang et al. [90] found that supplementation with saturated fatty acids during heat stress increased milk yield, milk fat content, and total milk solids, but also exacerbated thermal heat load. However, using extra fat during hot conditions can be tricky. Fatty acid oxidation generates more metabolic heat per unit of ATP captured (~2 kcal/g, or 13% on an energetic basis) than glucose [91]. While bypass fat generally does not reduce key physiological indicators of heat stress, such as respiratory rate and body temperature [87], it can improve energy balance and enhance lactation performance under both thermoneutral and heat-stress conditions [89,92]. These findings suggest that while bypass fat can support productivity during heat stress rather than directly mitigating physiological heat load, careful consideration of animal heat load and metabolic adaptations is critical to avoid exacerbating heat stress.

#### 5.2.2. Rumen-Protected Amino Acids and Micronutrients

Additionally, rumen-protected nutrients, including methionine, lysine, and choline, can help meet the nutritional requirements of high-yielding dairy animals and those under physiological stress, potentially improving milk quality, supporting metabolic function, reducing the negative effects of heat stress, and boosting immunity [93,94]. Their supplementation enhances antioxidative ability, leading to improved regulation of immunity and anti-inflammatory status in dairy cattle [94]. The rumen-protected methionine during heat stress can improve glucose concentration at calving and reduce the negative impact on calf birth and thermoregulation [95]. It maintains homeostasis in mammalian target of rapamycin (mTOR) and insulin signaling, while also supporting the whole-body antioxidant response and innate immune function [96]. Furthermore, Mesgaran et al. [97] reported that supplementing dairy cows with a rumen-protected zinc-methionine complex improved milk fat and protein content, reduced inflammation markers, and enhanced oxidative status under heat-stress conditions. Additionally, the use of functional fatty acids and amino acids may help support physiological resilience during stress conditions, such as transition periods and heat stress [98]. These nutritional components regulate and enhance compromised immunological, metabolic, and oxidative status in stressed animals.

Overall, these findings suggest that rumen-protected fat and amino acid supplementation can support production and metabolic stability during heat stress; however, evidence for their direct role in mitigating physiological heat stress remains limited and, in some cases, indirect. In addition, increasing unsaturated fatty acids can have physiological effects beyond energy provision, but may also have negative impacts on reproduction and immune function if not managed correctly [86,99]. Similarly, functional fatty acids and amino acids are hypothesized to alleviate stress during critical periods, but current evidence is inconsistent and context-dependent. Therefore, these strategies should be interpreted as supportive rather than definitive heat-stress mitigation approaches, and further studies are needed to confirm their direct effects on physiological responses to heat stress.

### 5.3. The Role of Polyphenols

Plant-derived polyphenols interact with saliva in ways that can affect digestion. Specific polyphenols found in non-centrifugal cane sugar have been shown to increase saliva secretion through various mechanisms, including enhanced intracellular calcium signaling and vasodilation [100]. Polyphenols show promise in mitigating heat-stress effects in cattle. These compounds can reduce oxidative stress and improve growth performance [101]. In bovine intestinal epithelial cells, tea polyphenols protect against heat-stress-induced damage by alleviating oxidative stress and inflammatory responses [102]. A supplement containing plant polyphenol extracts and electrolytes improved dairy cow performance, welfare indices, and enriched adipose tissue with oxidative stress response proteins during heat stress [103]. Polyphenols also play a role in regulating heat shock proteins and gut microbiota, which are essential for managing weaning stress in animals. Their immunomodulatory, antioxidative, and anti-inflammatory activities can help attenuate inflammation and improve digestibility [104]. These findings suggest potential benefits of incorporating polyphenols in cattle diets to combat heat stress. However, polyphenol–saliva interactions can also impact digestive processes. While some salivary proteins may prevent tannin-induced reduction in protein digestibility, polyphenols can inhibit salivary amylase, negatively affecting starch digestion [105].

### 5.4. Micronutrients for Oxidative Stress and Vasodilation

Heat stress in ruminants negatively impacts animal health, productivity, and welfare by inducing physiological imbalances, including oxidative stress and mineral disturbances [36,106]. Heat stress induces oxidative stress (OS) in dairy cows through multiple interconnected mechanisms. Hyperthermia is the primary driver, as elevated body temperature increases metabolic activity and mitochondrial respiration, leading to excessive production of reactive oxygen species (ROS) [24,107]. Additionally, heat stress activates the hypothalamic–pituitary–adrenal axis, elevating cortisol levels that promote catabolic processes and can impair antioxidant defenses. A further contributing factor is reduced feed intake, which limits the supply of dietary antioxidants such as vitamins A and E and trace minerals like selenium, weakening the cow’s ability to neutralize ROS [108,109]. Oxidative stress plays a role in disrupting metabolic homeostasis and impairing productivity, acting as a key mediator in the cascade of physiological imbalances triggered by environmental stressors such as heat [110]. Micronutrients, including minerals and vitamins, may improve ruminant performance under stressful conditions [111].

Any stressor, such as heat stress in ruminants, initiates a cascade of physiological and behavioral adaptations to maintain overall homeostasis, including thermal regulation, metabolic balance, and oxidative stability. The dissipation of heat from the core body part, especially the rumen, is extremely important. The blood circulation from the rumen towards the skin plays an important role in the release of heat produced during fermentation. Vasodilation, therefore, plays a crucial role in heat dissipation through increased blood flow through the skin, alongside increased respiration rates [112,113]. Niacin supplementation has shown potential to improve thermoregulation through its vasodilatory effects. It also has a positive impact on milk production and might counteract ketosis, although results vary [114]. Similarly, nitric oxide is involved in active cutaneous vasodilation during heat stress in humans; its relevance to cattle remains unclear. Cartwright et al. [115] observed that high immune responder cattle produced more nitric oxide in blood mononuclear cells under in vitro heat-stress conditions. Therefore, its relevance for dietary interventions in cattle should be considered indirect and requires further investigation.

Heat stress disrupts acid–base balance, increasing the risk of metabolic imbalance. To counter this, maintaining a positive dietary cation–anion difference through sodium and potassium salts helps stabilize blood pH and might in addition improve hydration, with documented benefits for water partitioning and intake under high temperature-humidity Index [116,117]. As heat stress increases reactive oxygen species and reduces antioxidant defenses, vitamin E with selenium enhances antioxidant capacity and immune function during hot seasons [118]. Under long-term heat stress, rumen-protected zinc improved milk components and lowered inflammation and lipid peroxidation, indicating better redox balance [119]. Additionally, chromium supplementation has been reported to enhance insulin sensitivity and reduce respiration rate, rectal temperature, and milk yield losses in some heat-stress studies; however, responses appear to be dose- and context-dependent [119,120].

The combination of heat exposure and redistribution of blood flow from the gut to the skin during heat stress can reduce intestinal perfusion, leading to localized hypoxia and oxidative stress that compromise epithelial integrity and tight junction function, thereby damaging the gut, allowing endotoxins to enter, and causing tissue damage and an immune response [121]. However, much of the supporting evidence originates from studies in monogastric species such as pigs. Direct evidence in cattle remains limited, and extrapolation should be made cautiously. To overcome this situation, Cronje and Qld [122] suggested using betaine to alleviate heat stress and enhance resilience by protecting the gut and liver against damage and the effects of endotoxins. Betaine plays a critical role in mitigating the negative effects of various metabolic processes, including osmoregulation, protein and lipid metabolism (as a methyl donor), and the antioxidant response [6]. It acts as an osmolyte, helping cells maintain water balance and reduce oxidative stress, thereby supporting gut integrity. It also serves as a methyl donor and enhances the performance of livestock animals [123,124]. Although cattle synthesize vitamin C, plasma levels decline under stress. Supplementation during heat stress has improved immune cell function and tempered cortisol levels, but controlled bovine heat-stress trials investigating the effect of vitamin C supplementation are still relatively few, so routine use should be framed as targeted rather than universal [118,119].

While these nutrients may help restore metabolic balance and improve performance, they can also have unintended side effects. For example, excessive mineral supplementation may disrupt electrolyte balance or interfere with rumen fermentation. Therefore, careful formulation and targeted application are essential to avoid adverse outcomes. This is a challenge in heat-stress situations where the level of feed intake reduction is associated with an increasing temperature-humidity index, and actual feed intake is difficult to predict by farmers. Ensuring a proper mineral balance, along with other strategies—such as adjusting dietary fiber, increasing energy concentration, and protein supply—is, however, essential to mitigate heat stress [36].

### 5.5. Probiotic Supplementation

Probiotic supplementation has shown promising effects in alleviating heat stress in goats and beef cattle, including improved rumen fermentation and performance parameters. Studies on beef bulls [125] and goats [126,127,128] demonstrated that probiotic supplementation improved growth performance, nutrient digestibility, and physiological responses under heat-stress conditions. Specifically, *Clostridium butyricum* and *Saccharomyces cerevisiae*, alone or in combination, enhanced rumen fermentation parameters, increased dry matter intake, and improved average daily gain in heat-stressed goats [126,127]. Supplementing *Saccharomyces cerevisiae* (yeast) in the diets of heat-stressed cattle can improve rumen health and digestion, thereby supporting milk production and potentially reducing stress indicators such as cortisol [129,130]. Additionally, probiotic supplementation led to increased total protein, albumin, and glucose levels in beef bulls [125]. In Hassani goats, probiotics reduced thermo-cardiorespiratory responses and increased total protein and globulin levels [128]. These benefits are partly attributed to the biological mechanisms by which probiotics modulate the gut and rumen microbiota, enhance barrier integrity, and reduce systemic inflammation [131,132]. Probiotics can suppress the proliferation of pathogenic microbes and promote the growth of beneficial bacteria which produce metabolites such as short-chain fatty acids that support epithelial health [133]. These studies suggest that probiotics can be an effective strategy for mitigating the adverse effects of heat stress on ruminant performance and welfare. However, most evidence comes from studies in goats and beef cattle, and data on lactating dairy cows remain limited. Therefore, the effectiveness of probiotics under heat stress should be interpreted with caution, and further controlled studies in cattle are required to confirm their role under commercial conditions.

## 6. Conclusions and Future Research Directions

Heat stress poses a significant challenge to cattle health, productivity, and welfare, particularly in the context of climate change. This review highlights the multifaceted physiological and microbial disruptions caused by heat stress and underscores the potential of nutritional strategies to mitigate its adverse effects. Key interventions include dietary adjustments to reduce metabolic heat production, supplementation with rumen-protected nutrients, polyphenols, antioxidants, and probiotics.

Nutritional strategies offer a practical and scalable approach to enhance resilience against heat stress. These interventions can improve energy balance, support immune function, reduce oxidative damage, and maintain rumen health. As the rumen is one of the major heat sources, rumen thermoregulation and microbial efficiency remain mechanistically distinct targets, suggesting that localized interventions may improve resilience. Future studies combining rumen physiology, microbial dynamics, and epigenetic programming are needed to optimize strategies that enhance productivity under heat stress.

While in vitro studies provide valuable mechanistic insights into rumen fermentation and microbial shifts under different nutritional changes, the absence of standardized in vitro models specifically designed to simulate heat-stress conditions further restricts their applicability. Therefore, future research should prioritize well-designed in vitro methods that incorporate heat stress to understand fermentation kinetics, metabolites, and the microbiome under heat-stress conditions.

Despite significant advancements in understanding the physiological and nutritional responses of cattle to heat stress, several knowledge gaps remain. In particular, the variability in reported rumen microbiome responses limits the ability to draw general conclusions about the relationship between microbiome and heat stress and their modulation by nutritional intervention. Most existing studies are either short-term or conducted under controlled conditions, and they are valuable for understanding specific effects; however, this may not fully capture the complexity of real-world farm environments. Additionally, there is a lack of comprehensive data on the long-term effects of nutritional interventions on animal health, productivity, and welfare during chronic heat stress. Addressing these gaps through standardized approaches and integrated research frameworks will be critical to improving animal resilience. By bridging current knowledge gaps and embracing innovation, the livestock industry can safeguard animal welfare and sustain productivity amid climate-induced heat stress.

## Figures and Tables

**Table 1:** Findings from different studies on changes in the microbiome associated with heat stress.

Heat Stress Duration	THI Condition	Feeding/Treatments	Animal Species, Breed and Type	Microbiome Change	Study
3 and 6 days 15 °C (control), 35 °C (heat stress), 60% humidity	Two weeks (58.78) and six days (86.86)	60% concentrate and 40% rice straw	Hanwoo steers (N = 4)	Planctomycetes decreased (3 days); Ruminococcaceae decreased; Lactobacillaceae, *Prevotella*, *Ruminobacter* increased (6 days)	[61]
4 weeks (Long term), 1 day (short term), 18–21 °C (control), 37 °C day/30 °C night (heat stress), 40–60% humidity	Control group (short term = 66.69, long term = 66.31), heat stress group (short term = 81.56, long term = 87.37)	Total mixed rations	Saanen dairy goats (N= 40)	Prevotella and Bacteroidetes increased; Succinimonas and Ruminobacter decreased	[65]
18 days per period with temperature fluctuation: 20 °C/55% humidity (control), 36 °C day/32 °C night/55% humidity (heat stress)	Pair-fed thermoneutrality (65.5), cyclic heat stress condition (night = 81.8, day = 87.2)	Total mixed ration	Lactating Holstein cows (N = 4)	Bacteroidetes increased; Firmicutes decreased	[34]
24 °C (thermoneutral), 34 °C (heat stress), 21 days per period	Thermoneutral (day = 68.7, night = 69.1), heat strees (day = 84.6, night = 79.6)	High energy and low energy with ad libitum and restricted intake	Nellore Heifers (N = 6)	Flavonifractor, Treponema, Ruminococcus decreased; Carnobacterium increased	[63]
2 weeks per temperature (20 °C, 28 °C and 33 °C)	Reference condition (66, 66.5), mild hot condition (76.4, 79.0), hot condition (83.2, 87.1)	50% hay and 50% concentrate	Lactating Holstein cows (N = 4)	*Clostridium coccoides—Eubacterium rectale*, *Streptococcus* increased; *Fibrobacter* decreased	[68]
2 months (Heat-sensitive and heat-tolerant cows)	May = 67.73, July = 84.16	Diet given according to the energy requirements of cows	Lactating Holstine cows (N = 14)	*Muribaculaceae, Rikenellaceae, Acidaminococcaceae, Christensenellaceae*, *Rikenellaceae_RC9_gut_group, Succiniclasticum, Ruminococcaceae_NK4A214_group*, and Christensenellaceae_R-7_group are higher in heat-tolerant cows. Prevotellaceae, Prevotella_1, Ruminococcaceae_UCG-014, and Shuttleworthia are higher in heat-sensitive cows.	[66]
120 days, temperatures: 26 °C, 30 °C, 34 °C, 38 °C), Relative humidity: 35%, 50%, 65%, 80%	69.17, 71.36, 73.08, 74.80, 75.99, 76.51, 78.30, 80.61, 80.62, 82.92, 83.52, 85.24, 86.42, 88.74, 89.33, 92.24, 95.74	Total mixed ration	Lactating Holstein cows (N = 18)	Firmicutes decreased; Bacteroidetes increased	[69]
60 days (summer)	Not available	Control group: Basal diet, treatment group: basal diet + *S. cerevisiae culture* in different dose	Lactating Holstine cows (N = 45)	Increased *Ruminococcus_gauvreauii_group, Butyrivibrio_2, Moryella, and Ruminiclostridium_6* fed with *S. cerevisiae culture* during heat stress	[64]

## Data Availability

No new data were created or analyzed in this study. Data sharing is not applicable to this article.
